# Prevalence of oral manifestations in coeliac disease and associated factors

**DOI:** 10.1186/s12876-025-03699-0

**Published:** 2025-03-03

**Authors:** Jani Manninen, Saana Paavola, Kalle Kurppa, Heini Huhtala, Teea Salmi, Katri Kaukinen, Camilla Pasternack

**Affiliations:** 1 Celiac Disease Research Center, Faculty of Medicine and Health Technology, Tampere University, Tampere, Finland; 2 Department of Internal Medicine, Tampere University Hospital, Wellbeing Services County of Pirkanmaa, Tampere, Finland; 3 Tampere Center for Child, Adolescent and Maternal Health Research, Tampere University and Tampere University Hospital, Wellbeing Services County of Pirkanmaa, Tampere, Finland; 4 The University Consortium of Seinäjoki, Seinäjoki, Finland; 5 Faculty of Social Sciences, Tampere University, Tampere, Finland; 6 Department of Dermatology, Tampere University Hospital, Wellbeing Services County of Pirkanmaa, Tampere, Finland

**Keywords:** Coeliac disease, Oral symptoms, Oral manifestations, Recurrent aphthous stomatitis, Aphthous ulcers, Dental enamel defects, Glossodynia, Gluten-free diet

## Abstract

**Background:**

Various oral manifestations are associated with coeliac disease in children, whereas data on adults are scarce. Moreover, possible individual factors predisposing to these manifestations remain unresolved. The aim of this study was to investigate these issues in a large cohort of adult coeliac disease patients both at diagnosis and while on gluten-free diet (GFD).

**Methods:**

This population-based study involved 873 adult patients with coeliac disease and 563 non-coeliac controls. Patients and controls were interviewed and structured questionnaires were used to assess the severity of gastrointestinal symptoms and quality of life at the time of the study. All participants were systematically asked about oral manifestations, including dental enamel defects, recurrent aphthous ulceration and glossodynia. Coeliac disease-related data were collected from medical records. Possible individual factors associated with oral manifestations were studied using logistic regression analysis.

**Results:**

Dental enamel defects were more common among patients than among non-coeliac controls (27% vs. 4%, *p* < 0.001). Prior to the coeliac disease diagnosis, 56% of the patients had experienced recurrent aphthous ulceration and GFD brought relief to 69% of them. While on GFD, coeliac disease patients had higher prevalence of recurrent aphthous ulceration than did the controls (17% vs. 13%, *p* = 0.040), but this significance disappeared after adjusting for gender. Glossodynia on GFD was more prevalent in the coeliac cohort than in the controls (14% vs 6%, *p* < 0.001). Oral manifestations at diagnosis and on GFD were associated with the presence of abdominal symptoms at the time of coeliac disease diagnosis, long diagnostic delay and female gender. At the time of the study, patients with oral symptoms had more severe gastrointestinal symptoms and poorer quality of life than those without these symptoms.

**Conclusions:**

Oral manifestations were more prevalent, at diagnosis and on GFD, in patients with coeliac disease than in the controls, and they were associated with long diagnostic delay, abdominal symptoms, female gender and impaired quality of life. A GFD was shown beneficial in relieving recurrent aphthous ulcerations in patients with coeliac disease.

## Introduction

Coeliac disease is a gluten driven immune-mediated enteropathy with an estimated global prevalence of 1–2% [1, 2]. It may develop at any age and the diverse clinical picture includes traditional gastrointestinal symptoms, but also symptoms originating from other organs such as skin, bones, liver and nervous system [3]. The disease may also manifest in the oral cavity, inducing both hard and soft tissue lesions [4, 5]. Dental enamel defects (DED) are the best-defined oral manifestations of coeliac disease*.* They appear symmetrically and chronologically in all four quadrants of the permanent teeth and are classified with the Aine classification [6]. Recent studies have connected DED with autoimmune process in coeliac disease [7], as was already suggested in the 1990’s [8]. Another oral manifestation commonly associated with coeliac disease is recurrent aphthous stomatitis (RAS) characterized by recurrent and painful mucosal ulcers [4]. Other oral symptoms, such as glossodynia, xerostomia and angular cheilitis, have also been reported in patients with coeliac disease [5]. A recent meta-analysis concluded that there is an increased risk for both DED and RAS in children with coeliac disease, but due to lack of research, the associations in adults are less clear [9].

A life-long gluten-free diet (GFD) is the treatment of choice for coeliac disease. GFD usually leads to histological and clinical remission and reduces the risk of long-term complications such as bone fracturs and malignancies [3]. There is some evidence that GFD may also alleviate soft tissue abnormalities of the oral cavity [10, 11], whereas already developed DED appear to be irreversible. The development of permanent dentition is completed in childhood, indicating that the risk for DED could be lower in patients with adult-onset coeliac disease, but more data are needed. Furthermore, there is some indication that the clinical phenotype of coeliac disease may affect the risk of associated oral manifestations [12, 13], but the relationship is not clear. Since more evidence is needed concerning oral manifestations in adult patients with coeliac disease, this study aimed to assess the prevalence of oral manifestations, particularly DED, RAS and glossodynia, in currently adult coeliac disease patients and to compare it to that in the non-coeliac control group. The effect of GFD on RAS was also evaluated, and, more importantly, the study aimed to identify the clinical factors associated with these oral manifestations in coeliac disease.

## Materials and methods

### Patients and controls

The study was conducted in Tampere University and Tampere University Hospital. Patients previously diagnosed with coeliac disease were recruited nationwide for the study in 2006–2010 via newspaper announcements and local coeliac disease patient societies. Each participant was interviewed by a physician or a study nurse with expertise in coeliac disease, blood samples were collected and structured and validated questionnaires were used to gather information about psychological well-being and gastrointestinal symptoms. Additionally, all coeliac disease-related relevant medical data, including histological and serological information, were collected from the medical records as available. Only participants who were at least 18 years of age at enrollment and who had small bowel biopsy-proven coeliac disease or skin biopsy-proven dermatitis herpetiformis (DH) were included in the present study. The final study cohort comprised altogether 873 patients.

Family members of the enrolled patients were also recruited and screened for coeliac disease using serum endomysial and tissue transglutaminase antibodies tests as a part of the original study in 2006–2010 and they were re-screened in 2017–2021. Further, the participating family members were interviewed by a physician or a study nurse. The study protocol is described in more detail elsewhere [14]. Adults not reporting coeliac disease and who remained screening-negative twice while on a gluten-containing diet comprised the non-coeliac control group for the present study (*n* = 563).

### Serological testing

Serum endomysial and tissue transglutaminase antibodies were tested at the time of the study from the patients with previously diagnosed coeliac disease, and the same antibody tests were used for screening the non-coeliac family members. Serum endomysial antibodies were measured by indirect immunofluorescence using the human umbilical cord as an antigen and considering titres 1: ≥ 5 positive. A commercial enzyme-linked immunosorbent assay was used for testing the tissue transglutaminase antibodies. In case of IgA deficiency was suspected IgG-class antibodies were measured [14].

### Clinical data

The coeliac disease-related data collected included the calendar year and age at coeliac disease diagnosis, the type, severity and duration of symptoms, positivity for coeliac disease autoantibodies at diagnosis and the results of histopathologic evaluation of the small bowel mucosa both at diagnosis and after a median of one year on a GFD. In addition, current long-term dietary adherence was inquired in the interview. Demographic data, presence of DED and oral symptoms, smoking status, and presence of comorbidities at the time of the study were likewise elicited both from the patients and non-coeliac controls. Presence of Sjögren’s syndrome in particular was assessed, as it is often associated with oral symptoms.

The severity of mucosal damage was further categorized as normal mucosal architecture, partial villous atrophy and subtotal/total atrophy according to the original pathology reports. Clinical presentation at diagnosis was divided into abdominal symptoms, malabsorption, dermatitis herpetiformis (DH), oral symptoms (see more detail below), other extraintestinal symptoms than DH or oral symptoms, and asymptomatic coeliac disease. Abdominal symptoms included reflux symptoms, abdominal distention or flatulence, constipation, nausea or vomiting, abdominal pain and diarrhoea, and extraintestinal symptoms other than DH e.g. musculoskeletal symptoms, neurological symptoms and liver enzyme abnormalities. Malabsorption denoted weight loss and/or characteristic blood tests abnormalities such as anaemia. Asymptomatic individuals were diagnosed by screening in risk-groups such as family members of coeliac disease patients and those with type 1 diabetes. Severity of symptoms was further categorized as mild, moderate or severe based on patients’ perceptions. Diagnostic delay referred to the duration of assumed coeliac disease-associated symptoms before diagnosis and was divided into three categories: less than 5 years, 5–10 years and over 10 years. Current long-term dietary adherence was further classified as strict (inadvertent gluten ingestion less than once in a month) or non-strict (occasional or frequent lapses) according to the interview.

Possible chronic comorbidities at the time of the study were categorized as immunological, gastroenterological, musculoskeletal, cardiovascular, psychiatric and neurological diseases. More specifically, presence of Sjögren’s syndrome, type 1 diabetes mellitus, autoimmune thyroid disease, asthma, allergies, lactose intolerance, inflammatory bowel disease, gastro-oesophageal reflux disease, cholecystolithiasis, hypertension, coronary heart disease, stroke, inflammatory arthropathies, degenerative disorders and osteopenia/-porosis was recorded. In addition, history of fractures and malignancies was elicited. Smoking was classified as current smoker, former smoker and never smoker.

### Oral manifestations

In the interview, both the coeliac disease patients and the non-coeliac controls were asked about the presence of DED. Further, occurrence of extraintestinal symptoms during the last month was systematically elicited with a multiple-choice questionnaire including separate items for recurrent aphthous ulcerations, referred in this study as RAS, and glossodynia. Also, among the coeliac disease patients, history of RAS prior the diagnosis of coeliac disease and the effect of GFD on RAS were elicited.

### Gastrointestinal symptoms and quality of life

The widely used structured and validated Gastrointestinal Symptom Rating Scale (GSRS) and the Psychological General Well-Being (PGWB) questionnaire were used to assess the severity of gastrointestinal symptoms and quality of life at the time of the study. Gastrointestinal Symptom Rating Scale (GSRS) is a 15-item questionnaire for evaluating severity of gastrointestinal symptoms in five different symptom groups: diarrhoea, indigestion, constipation, abdominal pain and reflux [15]. The symptoms are estimated on a seven-grade Likert scale, where higher number indicates increasing severity of symptoms. The PGWB assesses health-related quality of life and well-being using 22 items [16]. Each item is evaluated using a six-grade Likert scale and PGWB contains six sub-dimensions: anxiety, depressive mood, well-being, self-control, general health and vitality. Higher score indicates better psychological well-being.

### Statistical analysis

Descriptive data were presented as numbers and percentages, or as median values, interquartile ranges (IQR) and minimum and maximum values. The Pearson χ2-test and Mann–Whitney *U* test were used to compare coeliac disease patients to non-coeliac controls and to compare the GSRS and PGWB scores between those with and without oral symptoms at the time of the study. Further, binary logistic regression analysis was used to adjust for gender and age. Binary logistic regression analysis was also used to identify factors associated with oral symptoms in coeliac disease patients. After univariate analysis, multivariable binary logistic regression was conducted with significant risk factors identified in the univariate analyses to test for independent risk factors. Associations were expressed in odds ratios (OR) with 95% confidence interval (CI). *P*–value ≤ 0.05 was considered statistically significant. All the statistical analyses were performed with SPSS version 27 (IBM SPSS Statistics for Windows, Version 27. Armonk, NY: IBM Corp. USA).

## Results

### Prevalence of oral symptoms in coeliac disease patients at diagnosis and on gluten-free diet

The median age of the coeliac disease patients at diagnosis was 42 years, 53 patients (6%) were diagnosed in childhood and 76% were females. The patients were significantly more often female than the non-coeliac controls, whereas there was no significant difference in age at the time of the study, prevalence of Sjögren’s syndrome, or smoking habits (Table 1). At the time of the study, 12% (106/869) of the patients were seropositive to coeliac-related antibodies, and 96% (842/868) of the patients reported a strict adherence to GFD. DED were reported significantly more often among patients than controls (27% vs. 4%, *p* < 0.001). Altogether 56% of the patients reported having had RAS prior to the coeliac disease diagnosis. Of these, 69% reported GFD in relieving the ulcerations, while 31% continued to experience RAS despite the diet. At the time of the study, coeliac disease patients on GFD reported RAS more often than did those in the control group (17% vs. 13%, *p* = 0.040), but this significance disappeared after adjusting for gender. Glossodynia on GFD was more prevalent in the coeliac cohort than in non-coeliac controls (14% vs 6%, *p* < 0.001).

**Table 1:** Demographic data and presence of oral manifestations in coeliac disease patients and non-coeliac controls

	Coeliac disease patients, *n* = 873		Non-coeliac controls, *n* = 563	
	*n*	%	*n*	%
Females	665	76.2*	369	65.5
Age at the time of coeliac disease diagnosis, median (range), years	42 (0.5–81)		n.a	
Age at the time of the study, median (range), years	54 (18–89)		53 (18–97)	
Duration of gluten-free diet at the time of the study, median (IQR), years	9 (5–16)		n.a	
Sjögren syndrome at the time of the study	16	1.8	5	0.9
Smoking at the time of the study				
Current smoker	83	9.5	47	8.4
Former smoker	192	22	146	26
Never smoker	598	68.5	369	65.7
Dental enamel defects	185^a^	26.6**	23 ^b^	4.2
Recurrent aphthous ulcerations at diagnosis of coeliac disease	396^c^	56.3	n.a	n.a
Recurrent aphthous ulcerations at the time of the study	104^d^	16.7***	68^e^	12.5
Glossodynia at the time of the study	85^d^	13.7**	33^e^	6.1

*n.a*. not applicable, *IQR* interquartile range

^*^p < 0.001 between patients and controls

^**^p < 0.001 between patients and controls. Statistical significance persisted after adjusting for gender

^***^p < 0.040 between patients and controls, but statistical significance disappeared after adjusting for gender

^a^data available for 696 patients

^b^data available for 547 controls

^c^data available for 703 patients

^d^data available for 621 patients

^e^data available for 545 controls

### Factors associated with oral manifestations at the time of coeliac disease diagnosis

In coeliac disease patients, DED were associated with longer diagnostic delay, more severe coeliac disease symptoms, earlier diagnostic era and presence of abdominal symptoms at diagnosis (Table 2). More specifically, abdominal symptoms particularly associated with DED were nausea or vomiting (OR 1.86, 95% CI 1.04–3.30) and abdominal pain (OR 1.68, 95% CI 1.19–2.37). In multivariate analysis, long diagnostic delay, earlier diagnostic era and presence of abdominal symptoms at diagnosis remained significant independent risk factors. RAS at diagnosis was more common among female patients and among those with long diagnostic delay or abdominal symptoms at the time of the diagnosis (Table 2). Concerning abdominal symptoms, constipation (OR 1.79 95% CI 1.05–3.03) and diarrhoea (OR 1.36 95% CI 1.01–1.85) were especially associated with RAS. Female gender and abdominal symptoms at the time of diagnosis remained significant independent risk factors in multivariate analysis. Of note, neither DED nor RAS were associated with childhood diagnosis of coeliac disease, malabsorption, severity of small bowel damage at diagnosis (Table 2), or with seronegativity for coeliac-related antibodies at diagnosis (OR 1.21, 95% CI 0.62–2.36 and OR 1.04, 95% CI 0.58–1.89 respectively).

**Table 2:** Factors associated with dental enamel defects and recurrent aphthous ulcerations at diagnosis in coeliac disease patients

		Dental enamel defects			Recurrent aphthous ulcerations		
	*n*	%	OR	95% CI	%	OR	95% CI
Gender							
Male	172	23.0	1		47.1	1	
Female	531	27.7	1.28	0.85–1.93	59.3	**1.16**	**1.16–2.32**^d^
Age at diagnosis			1.00	0.98–1.01		0.99	0.98–1.00
Childhood diagnosis							
No	655	26.0	1		56.8	1	
Yes	49	32.7	1.38	0.74–2.58	47.7	0.70	0.38–1.28
Calendar period of coeliac disease diagnosis							
After 1999	349	21.8	1		53.9	1	
1990 – 1999	221	25.8	1.25	0.84–1.86	61.0	1.34	0.95–1.89
Before 1990	132	39.7	**2.37**	**1.53–3.65**^d^	54.5	1.03	0.69–1.54
Small bowel histology at diagnosis							
Normal	15	20.0	1		42.9	1	
PVA	186	26.6	1.45	0.39–5.35	54.3	1.58	0.53–4.75
SVA/TVA	362	26.0	1.40	0.39–5.08	59.1	1.93	0.66–5.67
Diagnostic delay							
< 5 years	372	22.9	1		53.5	1	
5 – 10 years	62	27.4	1.27	0.69–2.34	61.4	1.38	0.78–2.45
> 10 years	196	36.2	**1.92**	**1.31–2.80**^d^	62.8	**1.47**	**1.03–2.09**
Severity of symptoms at diagnosis							
Mild	186	23.7	1		51.1	1	
Moderate	347	26.4	1.16	0.76–1.75	58.5	1.35	0.94–1.93
Severe	128	35.9	**1.81**	**1.10–2.97**	61.7	1.54	0.98–2.44
Type of symptoms at diagnosis							
Abdominal symptoms							
No	126	17.7	1		45.2	1	
Yes	577	28.5	**1.85**	**1.13–3.03**^d^	58.8	**1.72**	**1.17–2.54**^d^
Malabsorption							
No	408	24.1	1		54.9	1	
Yes	298	29.9	1.34	0.96–1.88	58.3	1.15	0.85–1.56
Dermatitis herpetiformis							
No	568	27.2	1		56.5	1	
Yes	135	23.9	0.84	0.54–1.30	55.6	0.96	0.66 – 1.40
Other extraintestinal symptoms^b^							
No	598	25.7	1		55.5	1	
Yes	105	31.7	1.34	0.85–2.12	61.0	1.25	0.82–1.91
Asymptomatic^c^							
No	660	27.4	1		57.0	1	
Yes	43	14.3	0.44	0.18–1.07	46.5	0.66	0.35–1.22

*OR* odds Ratio, *CI* Confidence interval, *PVA* Partial villous atrophy, *SVA/TVA* Subtotal/total villous atrophy

^a^under 18 years of age at diagnosis

^b^other than oral symptoms or dermatitis herpetiformis

^c^screened in risk-groups such as patients with type 1 diabetes mellitus and relatives of coeliac disease patients

^d^Remained significant in multivariate regression analysis

### Factors associated with oral symptoms during GFD in coeliac disease patients

Presence of RAS during GFD was associated with female gender, younger age at the time of the study, long diagnostic delay and presence of other extraintestinal symptoms than DH at the time of diagnosis (Table 3). In addition, RAS were associated with lapses on GFD and shorter duration of GFD. Diagnosis of Sjögren’s syndrome, seropositivity to coeliac-related antibodies despite GFD or smoking status were not associated with presence of RAS (data not shown). When the presence of other comorbidities was assessed, decreased risk for RAS was seen in patients with cardiovascular disease (OR 0.54, 95% CI 0.32–0.92), particularly in those with hypertension (OR 0.40, 95% CI 0.21–0.78), but no other studied comorbidity was associated with RAS (data not shown). After multivariate analysis, age at the time of the study, long diagnostic delay and presence of other extraintestinal symptoms than DH at the time of diagnosis remained significant independent risk factors for RAS.

**Table 3:** Factors associated with recurrent aphthous ulcerations and glossodynia in coeliac disease patients on gluten-free diet (*n* = 621)

		Recurrent aphthous ulcerations			Glossodynia		
	*n*	%	OR	95% CI	%	OR	95% CI
Gender							
Male	153	11.1	1		5.9	1	
Female	468	18.6	**1.83**	**1.05–3.18**	16.2	**3.10**	**1.52–6.35**^f^
Age at diagnosis			0.99	0.98**–**1.01		1.00	0.95**–**1.02
Age at the time of the study			**0.98**	**0.97–0.99**^f^		1.00	0.99–1.02
Calendar period of coeliac disease diagnosis							
After 1999	296	17.2			12.8	1	
1990 – 1999	201	18.9	1.12	0.70–1.78	12.4	0.96	0.56–1.66
Before 1990	123	11.4	0.62	0.33–1.16	17.9	1.48	0.83–2.63
Childhood diagnosis^a^							
No	591	17.1	1		13.9	1	
Yes	29	6.9	0.36	0.08–1.54	10.3	0.72	0.21–2.42
Small bowel histology at diagnosis							
Normal	16	18.8	1		37.5	1	
PVA	165	15.8	0.81	0.22–3.05	13.3	**0.26**	**0.09–0.78**^f^
SVA/TVA	338	17.8	0.94	0.26–3.38	12.1	**0.23**	**0.08–0.67**^f^
Severity of symptoms at diagnosis							
Mild	158	17.7	1		11.4	1	
Moderate	316	15.8	0.87	0.53–1.45	15.2	1.39	0.78–2.49
Severe	111	19.8	1.15	0.62–2.13	17.1	1.61	0.80–3.22
Diagnostic delay							
< 5 years	303	13.2	1		13.9	1	
5 – 10 years	63	20.6	1.71	0.85–3.43	19.0	1.46	0.72–2.97
> 10 years	191	24.1	**2.09**	**1.30–3.34**^f^	15.2	1.11	0.67–1.86
Type of symptoms at diagnosis							
Abdominal symptoms							
No	116	14.7	1		6.0	1	
Yes	505	17.2	1.21	0.69–2.13	15.4	**2.84**	**1.28–6.34**^f^
Malabsorption							
No	355	14.6	1		13.5	1	
Yes	266	19.5	1.42	0.93–2.16	13.9	1.03	0.65–1.64
Dermatitis herpetiformis							
No	500	18.0	1		13.8	1	
Yes	121	11.6	0.60	0.33–1.09	13.2	0.95	0.53–1.71
Other extraintestinal symptoms^b^							
No	534	15.4	1		12.9	1	
Yes	87	25.3	**1.87**	**1.09–3.19**^f^	18.4	1.52	0.84–2.76
Asymptomatic^c^							
No	588	17.2	1		14.5	1	
Yes	33	9.1	0.48	0.14–1.61	0	-	
Follow-up small bowel histology^d^							
Normal	200	17.0	1		13.5	1	
PVA	134	17.2	1.01	0.57–1.81	17.2	1.33	0.73–2.43
SVA/TVA	20	25.0	1.63	0.55–4.78	15.0	1.13	0.31–4.12
Strict dietary adherence							
Yes	606	16.3	1		13.5	1	
No^e^	12	41.7	**3.66**	**1.14–11.76**	25.0	2.13	0.57–8.03
Duration of gluten-free diet			**0.97**	**0.95–0.99**		1.01	0.99–1.04

*OR* Odds ratio, *CI* Confidence interval, *PVA* Partial villous atrophy, *SVA/TVA* Subtotal/total villous atrophy

^a^under 18 years of age at diagnosis

^b^other than oral symptoms or dermatitis herpetiformis

^c^screened in risk-groups such as patients with type 1 diabetes mellitus and relatives of coeliac disease patients

^d^median of 1 year after the coeliac disease diagnosis

^e^occasional or frequent lapses on GFD

^f^Remained significant in multivariate regression analysis

Among coeliac disease patients on GFD, glossodynia was associated with female gender, prevalence of abdominal symptoms at the time of diagnosis and normal small bowel mucosal architecture at the time of the diagnosis (Table 3). Sjögren’s syndrome, seropositivity to coeliac-related antibodies despite GFD or smoking status was not associated with glossodynia (data not shown), but a significant association was seen in the presence of psychiatric comorbidities (OR 3.17, 95% CI 1.32–7.60), coronary artery disease (OR 2.68, 95% CI 1.14–6.30) and history of bone fracture (OR 1.68, 95% CI 1.04–2.72), while the other comorbidities studied showed no association (data not shown). Significant independent risk factors after multivariate analysis were presence of abdominal symptoms at the time of diagnosis, normal small bowel mucosal architecture at diagnosis and psychiatric comorbidity.

Patients with coeliac disease reporting RAS at the time of the study also had glossodynia more often than did those with no RAS (29.8% vs. 10.4%,* p* < 0.001). Furthermore, both RAS and glossodynia on GFD were associated with more severe gastrointestinal symptoms and with poorer quality of life when assessed with GSRS and PGWB at the time of the study (Table 4).

**Table 4:** Gastrointestinal Symptom Rating Scale (GSRS) and Psychological General Well-Being Questionnaire (PGWB) scores for coeliac disease patients (*n* = 621) with and without recurrent aphthous ulcerations and glossodynia on a gluten-free diet

	Recurrent aphthous ulcerations			Glossodynia		
	Yes, *n* = 104	No, *n* = 517	*p*-value	Yes, *n* = 85	No, *n* = 536	*p*-value
GSRS Total score	2.4 (1.7–2.9)	1.9 (1.5–2.4)	** < 0.001**	2.4 (1.8–2.8)	1.9 (1.5–2.5)	** < 0.001**
Diarrhoea	1.7 (1.0–2.3)	1.3 (1.0–2.3)	0.262	2.0 (1.0–2.7)	1.3 (1.0–2.3)	**0.002**
Indigestion	2.9 (2.0–3.8)	2.3 (1.8–3.0)	** < 0.001**	3.0 (2.0–3.6)	2.3 (1.8–3.2)	** < 0.001**
Constipation	2.0 (1.3–2.7)	1.7 (1.0–2.7)	**0.004**	2.0 (1.3–2.7)	1.7 (1.0–2.7)	0.006*
Abdominal pain	2.3 (1.7–2.9)	1.7 (1.3–2.3)	** < 0.001**	2.3 (1.7–2.7)	1.7 (1.3–2.3)	** < 0.001**
Reflux	1.5 (1.0–3.0)	1.5 (1.0–2.0)	**0.004**	1.5 (1.0–2.5)	1.0 (1.0–2.0)	**0.005**
PGWB Total score	101 (86–113)	107 (95–115)	**0.007**	98 (88–111)	107 (96–116)	** < 0.001**
Anxiety	24 (19–27)	25 (22–27)	**0.018**	23 (19–26)	25 (21–27)	**0.008**
Depressive mood	16 (14–18)	17 (15–18)	**0.032**	16 (14–18)	17 (15–18)	** < 0.001**
Well–being	17 (14–19)	18 (15–20)	0.039*	16 (14–19)	18 (15–20)	**0.009**
Self–control	15 (13–17)	16 (14–17)	**0.009**	15 (13–16)	16 (14–17)	**0.001**
General health	12 (10–15)	13 (11–15)	0.092	12 (9–14)	13 (11–15)	** < 0.001**
Vitality	17 (14–20)	18 (16–20)	**0.005**	17 (15–19)	19 (16–21)	** < 0.001**

The values are given as medians with interquartile range

In the GSRS, a higher score indicates more severe symptoms, and in the PGWB, a higher score indicates better quality of life

^*^Significance disappears after adjusting for gender and age at the time of the study

## Discussion

This study showed that both hard and soft tissue oral manifestations were more prevalent in our adult coeliac patients than among the non-coeliac controls, and that GFD is beneficial for coeliac disease patients with RAS. More importantly, the study showed that oral manifestations were associated with abdominal symptoms and also with long delay in diagnosis and with female gender.

Risk of DED was found to be greater in patients compared to non-coeliac controls. This is in line with most earlier studies reporting increased risk of DED in adult coeliac disease patients, the reported prevalences ranging from 14 to 83% in patients and from 0 to 9% in controls [8, 10, 17–19], although contrasting findings have also been reported [20]. Furthermore, 56% of the patients reported that they had suffered from RAS prior to diagnosis, which was decidedly more than in the controls (13%) at the time of the study. This over-representation of RAS with relatively high percentage is generally in line with earlier reports [10, 11, 17, 18], and the observed association is further corroborated by the increased prevalence of coeliac disease among patients with RAS [4, 21, 22]. In addition, the majority of patients here (69%) reported reduction of RAS with the introduction of GFD, which is again in accord with earlier studies reporting response rates from 67 to 89% [10, 11]. The present study also found no difference in the prevalence of RAS between patients on GFD and controls, which contrasts with previously reported findings [17, 23], but reinforces the beneficial effect of GFD in coeliac disease in this cohort. However, the presence of glossodynia was found to be higher among treated coeliac disease patients than among the controls. A comparable finding was reported by Van Gils et al. [23], and an increase in glossodynia has also been shown in paediatric coeliac disease patients [24–26].

There was a significant association between the oral manifestations studied and the presence of abdominal symptoms at coeliac disease diagnosis. Furthermore, the oral manifestations were also linked to more severe gastrointestinal symptoms at the time of the study among patients on GFD as measured with GSRS, thus reinforcing the relationship of these symptoms, both of which originate from the gastrointestinal tract. Oral manifestations have also been previously associated with the classic phenotype of coeliac disease both in children and adults [12, 13, 27–29], although contradictory results have also been reported, especially in children [30, 31]. Moreover, the present study showed an association between the presence of DED and earlier diagnostic era, i.e. diagnosis before the year 1990. As the clinical picture of coeliac disease has changed over the decades and the classic phenotype and more severe disease have been more common in earlier times [3], this association is not surprising and supports the previous observation. Previously an association has been shown between gastrointestinal symptoms, degree of mucosal damage and coeliac disease related antibodies [32]. In this study duodenal damage or seropositivity were not associated with other studied oral symptoms, but glossodynia on GFD was more often seen in patients with normal mucosal architecture at diagnosis. The latter is an interesting observation and analogous with the fact that patients with DH, who have symptomatic skin lesions, also evince more often normal mucosal architecture at diagnosis than patients with other phenotypes of coeliac disease [33].

A long delay in diagnosing coeliac disease was associated with increase in risk of both DED and RAS on GFD in the current study. In paediatric populations, older age at diagnosis has been connected with increased risk and severity of DED, and prolonged gluten exposure has been proposed as the cause [20, 31]. In adults this has not been shown before, and the association is more difficult to explain as permanent dentition develops in childhood. However, it has been proposed that adult patients with DED have already had mild unrecognized coeliac disease in childhood [8], and this long history of coeliac disease-related symptoms observed in adult patients with DED could be associated with this unrecognized disease. The relationship between long diagnostic delay and RAS on GFD is by contrast not surprising as diagnostic delay is in general associated with poorer resolution of symptoms on GFD [34–36]. Of note, abdominal symptoms were also more severe in patients with oral symptoms on GFD, indicating incomplete recovery from symptoms in general in this patient group.

There was also an association between female gender and RAS at diagnosis and glossodynia on GFD. Gender has not been linked to oral symptoms in adult patients in the few previous studies [20, 23], but an association with gender and the clinical picture of coeliac disease at diagnosis has been reported [11, 36–38]. Furthermore, higher frequency of abdominal symptoms and poorer clinical recovery on GFD have been reported among females with coeliac disease than among males [11, 39]. Also, younger age at diagnosis and other extraintestinal symptoms than DH were associated with RAS on GFD. These findings have not been observed before and should be replicated prior to drawing any firm conclusions.

Ongoing oral manifestations on GFD were also associated with impaired quality of life. The impact of oral health on quality of life has been studied in other diseases [40, 41], but data in coeliac disease are scant. However, as an indirect comparison, impaired quality of life has been linked to persistent symptoms in coeliac disease [35, 42]. In addition, Van Gils et al. used an oral health-related questionnaire to measure the impact of oral symptoms on well-being [23], and showed decreased well-being in coeliac disease patients compared to controls, but did not study the impact of oral symptoms on well-being within the coeliac disease cohort. Of note, psychiatric disorders were more prevalent among patients with glossodynia in the present study, which may in part explain the association observed.

The strengths of this study are the large and well-defined nationwide cohorts of coeliac disease patients and non-coeliac controls and the use of validated questionnaires in the assessment of gastrointestinal symptoms and quality of life. In addition, coeliac disease-related data were confirmed meticulously from the medical records. The main weakness is the use of self-reported and partially retrospective data in the assessment of oral manifestations, as due to the study design, it was not possible to conduct systematic clinical examination of the dentation or oral mucosal. Furthermore, the recruitment of patients via coeliac societies and the use of non-coeliac controls among at-risk family members may have caused selection bias [43].

## Conclusions

Oral manifestations are associated with coeliac disease, also in adult patients, and the clinical phenotype with traditional abdominal symptoms and female gender seems to predispose patients to oral involvement. Furthermore, as long delay to diagnosis was also associated with oral manifestations and introduction to GFD seems to benefit these patients, early diagnosis of coeliac disease should be pursued. Once on GFD, ongoing oral symptoms are linked to persistence of gastrointestinal symptoms and impaired quality of life. These findings would speak for better recognition of these patients as they may need more careful follow-up.

## Data Availability

The datasets used and/or analyzed during the current study are available from the corresponding author on reasonable request.

## Abbreviations

CI: Confidence interval
DED: Dental enamel defects
DH: Dermatitis herpetiformis
GFD: Gluten-free diet
GSRS: Gastrointestinal Symptom Rating Scale
IQR: Interquartile range
OR: Odds ratio
PGWB: Psychological General Well-Being
RAS: Recurrent aphthous stomatitis
